# Free Energies of Hydrated Halide Anions: High Through-Put Computations on Clusters to Treat Rough Energy-Landscapes

**DOI:** 10.3390/molecules26113087

**Published:** 2021-05-21

**Authors:** Diego T. Gomez, Lawrence R. Pratt, David M. Rogers, Susan B. Rempe

**Affiliations:** 1 Department of Chemical & Biomolecular Engineering, Tulane University, New Orleans, LA 70118, USA; dgomez1@tulane.edu (D.T.G.); lpratt@tulane.edu (L.R.P.); 2 National Center for Computational Sciences, Oak Ridge National Laboratory, Oak Ridge, TN 37830, USA; rogersdm@ornl.gov; 3 Center for Integrated Nanotechnologies, Sandia National Laboratories, Albuquerque, NM 87185, USA

**Keywords:** ion hydration, physical cluster theory, halides, Hofmeister series, specific ion effects

## Abstract

With a longer-term goal of addressing the comparative behavior of the aqueous halides F${}^{-}$, Cl${}^{-}$, Br${}^{-}$, and I${}^{-}$ on the basis of quasi-chemical theory (QCT), here we study structures and free energies of hydration clusters for those anions. We confirm that energetically optimal ${\left(H_{2}O\right)}_{n}X$ clusters, with X = Cl${}^{-}$, Br${}^{-}$, and I${}^{-}$, exhibit *surface* hydration structures. Computed free energies, based on optimized surface hydration structures utilizing a harmonic approximation, typically (but not always) disagree with experimental free energies. To remedy the harmonic approximation, we utilize single-point electronic structure calculations on cluster geometries sampled from an AIMD (*ab initio* molecular dynamics) simulation stream. This *rough-landscape* procedure is broadly satisfactory and suggests unfavorable ligand crowding as the physical effect addressed. Nevertheless, this procedure can break down when n≳4, with the characteristic discrepancy resulting from a relaxed definition of clustering in the identification of ${\left(H_{2}O\right)}_{n}X$ clusters, including ramified structures natural in *physical cluster theories.* With ramified structures, the central equation for the present rough-landscape approach can acquire some inconsistency. Extension of these physical cluster theories in the direction of QCT should remedy that issue, and should be the next step in this research direction.

## 1. Introduction

Ions exert specific effects on molecules. Hofmeister devised a ranked list of salts to classify their influence on protein precipitation and swelling [1,2]. Later works identified ion-specific effects on molecules more generally, in both aqueous and non-aqueous solutions, at interfaces, and in confined settings such as biological transport proteins and ionomers [3,4,5,6] with little or no solvent. Indeed, molecular-level mechanisms of ion-specific effects is a topic of current research [7,8,9]. Important aspects of those mechanisms in aqueous solution involve the local ion hydration properties, and exchange of hydrating water molecules for molecular ligating groups [3,5,10,11,12]. As a step toward enhancing our understanding of ion-specific effects, this paper studies the structures and free energies of hydration clusters of the anions F${}^{-}$, Cl${}^{-}$, Br${}^{-}$, and I${}^{-}$ in the dilute gas-phase, with the longer term goal of addressing the comparative behavior of this series of ions in liquid water on the basis of quasi-chemical theory (QCT) [12,13,14,15]. Accurate characterization of gas-phase clusters can indeed helpfully inform QCT applications, and has been successful for cations [12]. Because our analyses here will be limited to gas phase systems, we restrict our work to *physical cluster theories* [16,17,18,19,20,21,22], progenitors of molecular QCT [23,24].

Common to all these theories is the study of associative equilibria

(1) $$nW+X⇌W_{n}X\;.$$

Here X ≡ F${}^{-}$, Cl${}^{-}$, Br${}^{-}$, or I${}^{-}$, and the dissolving medium provides ligand $W\equiv H_{2}O$ molecules. Equation (1) then directs attention to

(2) $$\begin{array}{c} K_{n}^{\left(0\right)}=\frac{\rho_{W_{n}X}}{\rho_{W}{}^{n}\rho_{X}}\;, \end{array}$$

where $\rho_{W_{n}X}$ is the number density of W${}_{n}$X species. Then $K_{n}^{\left(0\right)}$ is the traditional equilibrium constant appropriate for the case that the dissolving medium is an ideal gas. That ideal gas restriction is indicated by the superscript notation. In QCT, slightly more subtle considerations arrive also at Equation (2), which here serves as our starting point [25]. These theories all require logical definition of formed W${}_{n}$X clusters for counting. Such definitions amount to defining proximity of a W ligand to an X ion. Although judgement might be required to establish a physically effective proximity definition, here we will assume that, under the standard temperatures considered below and the low density conditions of interest, practical molecular simulations of W${}_{n}$X systems encounter clustered configurations entirely. This definition will be reconsidered in subsequent QCT developments.

**Figure 1:**
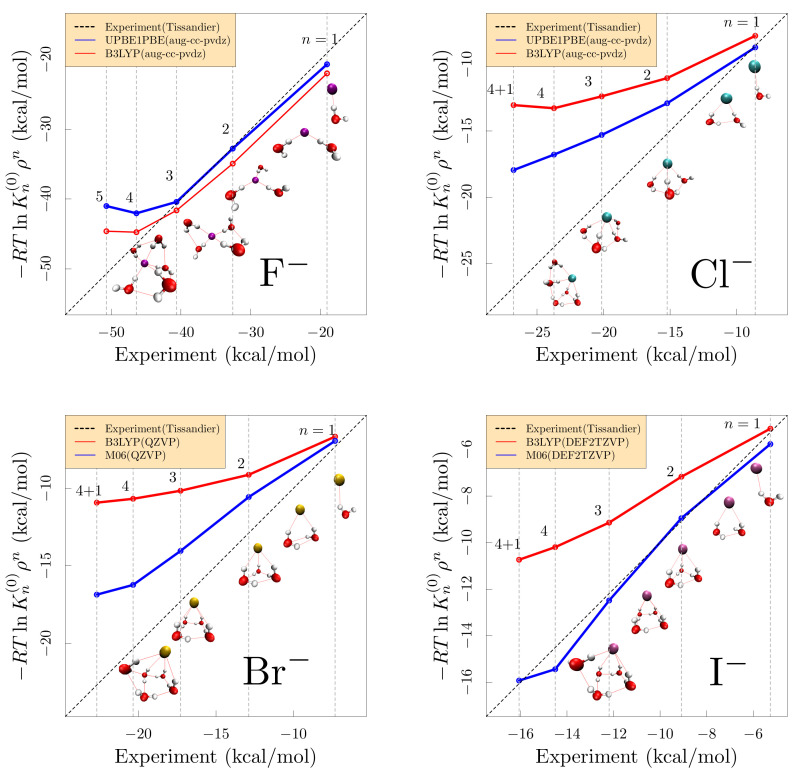
Cluster 1≤n≤5 free energies from harmonic approximation based upon energy optimized structures (see Section 4.1), against experimental values for ${\left(H_{2}O\right)}_{n}X$, X = F${}^{-}$ Cl${}^{-}$, Br${}^{-}$, and I${}^{-}$. The indicated electron density functionals and basis sets are merely typical choices in current practice; comparisons of these results suggest typical variability and should not suggest physical discriminations. The inset molecular graphics illustrate that asymmetric, *surface-hydrated* structures are optimal geometries for clusters involving Cl${}^{-}$ and heavier halides. For n=5 and Cl${}^{-}$, Br${}^{-}$, and I${}^{-}$, optimizations pushed a water molecule into a distinct outer shell, and those structures are denoted as 4+1 clusters. For all of these ions, the harmonic approximation for the n=1 case produces an accurate estimate for the experiment, which indicates that the dissimilarity for the other cluster sizes n≥2 arises from interactions between the ligands.

The computational study of clusters benefits from a good experimental basis [26]. Widely available chemical software enables straightforward numerical evaluation of $K_{n}^{\left(0\right)}$ [26] under assumptions of harmonic motion on the potential energy surface near an optimized cluster geometry (Figure 1). The assumption of harmonic motion is valid for clusters with strong interactions that limit atomic displacements to small distances away from an optimized geometry [27]. A challenge common to harmonic and anharmonic estimates of the free energy is that more than one low energy structure may be relevant [28,29,30]. Similar to earlier results found for simple cations [28,31], harmonic approximations (Figure 1) are satisfactory for W${}_{n}$F${}^{-}$ and n≤4, but not for n=5 in that case. From the disagreement with experiment (Figure 1), we see that the harmonic treatment is not satisfactory for W${}_{n}$Cl${}^{-}$. For those challenging cases, previous work provided a simple fix of that discrepancy [15], making use of cluster configurations sampled from dynamical simulations. The goal of this paper is to test whether inaccuracies of the harmonic treatment are satisfactorily resolved also for the rest of the halide series (Br${}^{-}$, I${}^{-}$) by analysis of structures from dynamical simulations. A similar approach was applied earlier to correct harmonic approximations applied to compute chemical equilibria and free energies of H${}_{2}$ hydration clusters [32].

The essential feature of the computational approach here is the deployment of standard electronic structure computations for cluster geometries sampled from AIMD (*ab initio* molecular dynamics) simulations of $W_{n}X$ clusters. A detailed accounting from the single-point energy procedures defined with Equation (5) below indicates more than 10${}^{3}$ calculations for n=5 with standard modern electronic structure methods for single geometries from the AIMD canonical simulation stream. Thus, we implement a high-throughput algorithm, described below, to carry-out the calculations efficiently.

We emphasize the potential utility, in the longer term, of this work to QCT applications to bulk solutions. QCT puts a high premium on understanding the structure and energetics of small clusters that can serve to fill-out an inner shell, then treating W${}_{n}$X as a primitive chemical constituent of the solution. Therefore, we limit *n* to values smaller than the historical interest [33,34,35,36,37,38,39,40,41,42,43]. Specifically, we do not attempt to push *n* to values large enough to suggest an incipient droplet of liquid. Instead, we seek a sharper understanding of $W_{n}X$ structures, energetics, and particularly rough landscape effects for small values of *n* [15]. Nevertheless, the present results are broadly consistent with preceding simulation work on such systems, including on larger values of *n*, studied with empirical intermolecular force fields [33,34,35,36,37,38,39,40,41,42,43]. Though this work purposely avoids taking the large cluster limit, an alternative QCT model which does seek that limit appeared recently [44].

## 2. Results

**Figure 2:**
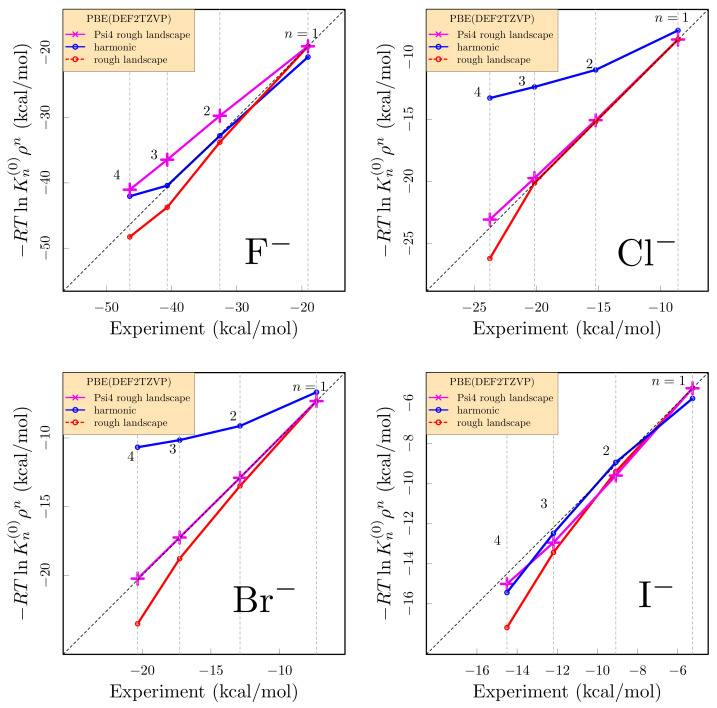
Harmonic (blue, Figure 1) and rough landscape (red) free energies, against experimental results [45]. Results labeled Psi4 (violet) used the PBE functional [47] and an DZVP-MOLOPT-SR-GTH basis for CP2K/AIMD and DEF2TZVP basis for Gaussian/single-point calculations. For comparison, M06/CP2K and B3LYP/Gaussian for F${}^{-}$, Cl${}^{-}$, and Br${}^{-}$ clusters and M06/Gaussian for I${}^{-}$ results are also shown in red.

We evaluated structures and formation free energies of $W_{n}X$ clusters in gas phase. We compared: (i) the experimental values determined from mass spectrometry [45]: (ii) the quantum-mechanical rigid rotor harmonic oscillator approximation (Figure 1), and (iii) the rough-energy landscape procedure proposed in Ref. [15]. Figure 2 summarizes all results graphically. The free energies decrease by about 5 kcal/mol for each water added. Free energies follow the expected size trend—smaller anions bind water more strongly. Associated with the energetic trend, and correlated with increasing anion polarizability [46], the first solvation shell becomes more asymmetric with increasing anion radius.

**Figure 3:**
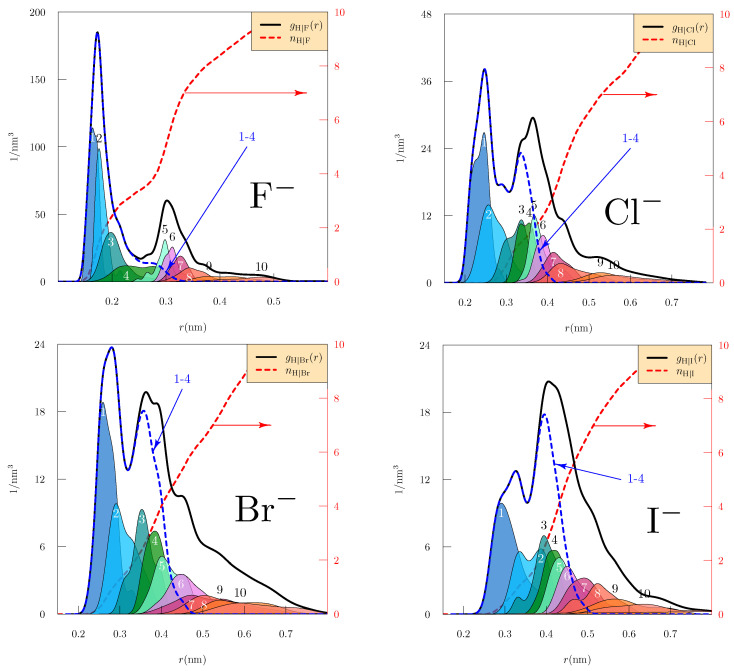
Radial distributions of H atoms from the indicated ions observed from 5 ps of AIMD trajectory of the clusters W${}_{5}$X, after 5 ps of equilibration as discussed in Section 4. The integer labeled curves are distributions of neighborship-ordered H atoms, e.g., the curve labeled ‘1’ is the distribution of the nearest H atom. The distributions and the vertical axes have units of 1/nm${}^{3}$, and those distributions are normalized as $4\pi \int_{0}^{\infty }\rho^{\left(j\right)}\left(r\right)r^{2}dr=1$ The average included number of H-atoms, $n_{H|X}\left(r\right)$ is the red-dashed curves and right-side axes.

Structures of the W${}_{5}$X clusters should sharpen our observations. Densities of H-atoms radially from the central ion ($n_{H|X}\left(r\right)$, Figure 3) evaluated within simulations including 5 waters show a distinct step near $n_{H|X}\left(r\right)=4$ for the F${}^{-}$ case. Even though more water molecules are available, a sharply defined 4-coordinate structure with H-bond donation for that inner-shell is observed. This is a natural rationale for the satisfactory performance of primitive QCT theory for F${}^{-}$(aq) [48,49]. Structures observed for the other ions (Figure 3) are individually more complicated.

**Figure 4:**
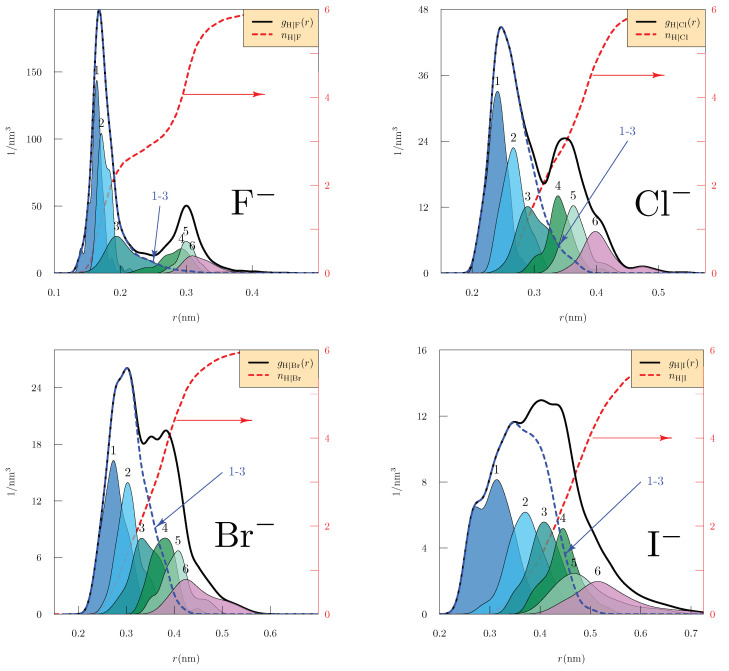
Radial distributions of H atoms from the indicated ions observed from 5 ps of AIMD trajectory of the clusters W${}_{3}$X after 5 ps of aging, as discussed in Section 4. The distributions and the vertical axes have units of 1/nm${}^{3}$. The integer labeled curves are distributions of neighborship-ordered H atoms, as discussed in Figure 3, and normalized as $4\pi \int_{0}^{\infty }\rho^{\left(j\right)}\left(r\right)r^{2}dr=1$. The average included number of H-atoms ($n_{H|X}\left(r\right)$, red-dashed curve), which are gauged by the right-side axes.

It might be guessed that these structural characteristics would be simpler for n=3 clusters, i.e., for W${}_{3}$X clusters (Figure 4) for which splitting of the inner-shell should not be important. Note here the distinct step near $n_{H|X}\left(r\right)=3$ for the F${}^{-}$ case, reinforcing the picture of classic H-bond donation to that ion. For the Cl${}^{-}$, Br${}^{-}$, and I${}^{-}$ ions, consider W${}_{3}$X (Figure 4), in contrast to W${}_{5}$X (Figure 3). The H-atom radial layering might be slightly simpler for n=3 than for n=5, but the distinction between the 3rd and 4th nearest H-atoms is qualitatively less striking for the heavier three halides than for F${}^{-}$. This suggusts that those heavier three halides rely less on simple H-bond donation structures than does F ${}^{-}$; an explicit examination of the role of dipole-donation structures for WCl${}^{-}$ from the cluster dynamics was given recently [15].

**Figure 5:**
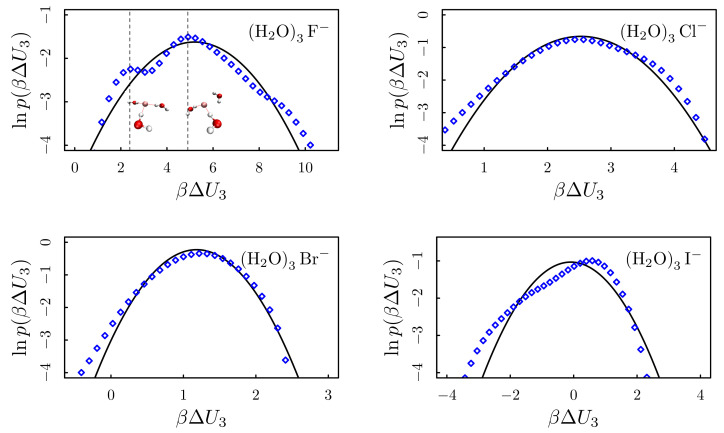
Distributions of $\beta \Delta U_{3}$ of Equation (5) for halide-water clusters, observed during 10 ps of CP2K trajectory after 5 ps aging. Split-shell geometries are not expected to be important for these n=3 cases (Figure 1). Nevertheless, $W_{3}F^{-}$ shows a multimodal distribution, the maxima located by dashed lines. The lower energy mode can be associated with the optimized structure (Figure 1) while the higher energy mode describes configurations that can flip a water molecule to offer a different H atom for coordination with the ion. The solid lines are the Gaussian model distribution with the sample mean and variance. These $\beta \Delta U_{3}$ reflect contributions from crowding of ligands, mostly unfavorable here. The estimated mean for $W_{3}I^{-}$ is near zero, consistent with the realized performance of the harmonic approximation of that case (Figure 1).

The energies (Figure 5) required for the rough energy landscape method of Equations (4)–(6) provide an energy-based view of water’s interaction with the anion cluster. These energies are mostly unfavorable with magnitudes extending to roughly 5 $k_{B}T$ or about a typical H-bond energy. The observed distributions of these energies are interesting, but not troublesome for the theoretical procedure Equation (6).

## 3. Discussion

We confirm (Figure 1) that energetically optimal ${\left(H_{2}O\right)}_{n}X$ clusters with X = Cl${}^{-}$, Br${}^{-}$, and I${}^{-}$ exhibit *surface* hydration structures. As discussed in the Introduction, Section 1, for those cases, free energies based on harmonic approximation of the potential energy surface and optimal structures typically (but not always) disagree with experimental free energies. As noted above, goal of this paper is to test whether inaccuracies of the harmonic treatment are satisfactorily resolved consistently for the whole of the halide series by our analysis of structures from dynamical simulations.

For the Cl${}^{-}$ and Br${}^{-}$ ions (Figure 2), the rough-landscape procedure (Equation (6)) makes the distinctive correction anticipated *except* for the n≳4 cases. That exceptional n≳4 behavior is exhibited also by I${}^{-}$, though the I${}^{-}$ case is unusual in that the harmonic approximation is accurate. We attribute the exceptional n≳4 behavior to the influence of split-shell structures, including 4+1 structures for n=5, in the data stream for these physical clusters. Figure 1 shows 4+1 structures, where 4 waters form direct hydrogen bonds to the anion and one additional water lies outside the inner-shell. Split-shell clusters have been documented for simple cations [50,51,52,53,54,55], and used to rationalize discrepancies between computed and experimental gas phase cluster data [56]. The F${}^{-}$ case is simpler overall, though n≥4 begins to incur the characteristic error of the harmonic approximation in this application. Still the rough-landscape procedure (Equation (6)) improves the comparison with experiment for the F${}^{-}$ case, too.

## 4. Materials and Methods

### 4.1. Software and Procedures

Molecular dynamics trajectories of the isolated ${\left(H_{2}O\right)}_{n}X$ for 2≤n≤5 and X = F${}^{-}$ Cl${}^{-}$, Br${}^{-}$, and I${}^{-}$ were obtained using CP2K [57,58]. The M06 [59] functional was utilized as our standard case with pseudopotentials proposed by Goedecker, Teter and Hutter (GTH [60]) in the Gaussian and plane wave schemes [61]. Molecularly optimized DZVP-MOLOPT-SR-GTH [62] basis sets were obtained from the CP2K website. Temperatures were set at 300 K with the Nosé-Hoover thermostat, a time step of 1 fs for 15 ps total of trajectory with the last 10 ps used for analysis.

Our initial assessment of cluster free energies (Figure 1) applied the harmonic approximation to geometry-optimized structures. As initial conditions for the geometry optimization, $N_{s}$ = 20 uniformly spaced configurations were extracted from the last 10 ps of CP2K trajectories and those sampled configurations were optimized using Gaussian09 [63] with the B3LYP [64,65] functional for all ions, in addition to PBE [47] for F${}^{-}$ and Cl${}^{-}$ utilizing the aug-cc-pvdz basis set [66,67], and M06 for Br${}^{-}$ and I${}^{-}$ utilizing QZVP [68] and DEF2TZVP [68] basis sets, respectively. Finally, $-RT\ln K_{n}^{\left(0\right)}$ (Figure 1) was evaluated in the harmonic approximation for the lowest energy structure. The symmetry number—the n! of Equation (3)—was assigned as discussed by Muralidharan, et al., [49]. For ${\left(H_{2}O\right)}_{5}{\mathrm{Br}}^{-}$, ${\left(H_{2}O\right)}_{5}{\mathrm{Cl}}^{-}$ and ${\left(H_{2}O\right)}_{5}I^{-}$ (Figure 1), optimizations push a water molecule into an outer solvation shell, and those structures thus can be identified as 4+1 clusters.

For the rough landscape treatment, structures from 2≤n≤5 clusters are sampled every 0.1 ps of the last 10 ps of the AIMD trajectory. Each sampled configuration is decoupled, according to the right-hand side of Equation (5), and the structures subjected to single-point calculations using Gaussian09 with the B3LYP functional and aug-cc-pvdz basis set for F${}^{-}$, Cl${}^{-}$, Br${}^{-}$ and M06 functional and DEF2TZV basis set for I${}^{-}$ configurations. Using the resulting thermal averaging in Equation (6), and $K_{1}^{\left(0\right)}$ from experiment, the resulting $K_{n}^{\left(0\right)}$ produce the results in Figure 1 in a step-wise fashion.

To confirm our results, and to investigate the functional dependence of the cluster free energy, additional AIMD trajectories for 2≤n≤5 clusters of all halides were carried out independently. These were also done with CP2K, this time using the PBE [47] functional with GTH psuedopotentials and molecularly optimized DZVP-MOLOPT-SR-GTH basis set, using the Nosé-Hoover thermostat at 300 K. Structures from AIMD were again sampled every 0.1 ps from the last 10 ps of 15 ps of the CP2K trajectory. Single-point energy computations of the energy differences indicated by Equation (5) for 2≤n≤5 clusters, used the PBE functional and all-electron DEF2TZVPD [69] basis set, as implemented by Psi4 [70].

### 4.2. Theory

Classic statistical thermodynamics [13,15,71] anchors our analysis of

(3) $$\begin{array}{ccc} K_{n}^{\left(0\right)} & = & \frac{Q\left(W_{n}X\right)/n!}{Q\left(X\right){\left[Q(W)/V\right]}^{n}}\;. \end{array}$$

Here *V* is the system volume and $Q\left(W_{n}X\right)$ are single molecule (cluster) canonical partition functions, *configurational integrals* in the traditional classical-limit analysis [71]. In this ratio, symmetry numbers associated with W molecules can be cancelled on top-and-bottom, and the n! reflects the permutation symmetry of the *n* W ligands. Each $Q\left(W_{n}X\right)$ is proportional to *V*, so the ratio Equation (3) is independent of *V*.

Our scheme for evaluating $K_{n}{}^{\left(0\right)}$ proceeds by step-wise addition of waters (n−1→n) according to

(4) $$\begin{array}{c} \frac{K_{n}{}^{\left(0\right)}}{K_{n-1}{}^{\left(0\right)}K_{1}{}^{\left(0\right)}}=\frac{Q\left(W_{n}X\right)/Q\left(X\right)}{n\left[Q\left(W_{n-1}X\right)/Q\left(X\right)\right]\left[Q\left(WX\right)/Q\left(X\right)\right]}\;. \end{array}$$

The numerator on the right of Equation (4), involves integration carried over configurations of a $W_{n}X$ cluster, i.e., integrated over clustered configurations, canonically weighted as $\exp\left[-\beta U(W_{n}X)\right]$. Here β=1/kT, and $U(W_{n}X)$ is the electronic energy of the $W_{n}X$ cluster in a given geometry. Because of the divisor in the combination $Q\left(W_{n}X\right)/Q\left(X\right)$, that combination is independent of the system volume. The combined denominator treats configurations of $W_{n-1}X$ and WX, but independently of each other. Of course, exactly the same integrations are expressed in numerator and denominator of Equation (4). Introducing

(5) $$\begin{array}{c} \Delta U_{n}=U\left(W_{n}X\right)-U\left(W_{n-1}X\right)-U\left(WX\right)+U\left(X\right)\;, \end{array}$$

then the denominator of Equation (4), expressing the same integrations as the numerator, is merely missing the factor $\exp\left[\beta \Delta U_{n}\right]$. Therefore

(6) $$\begin{array}{c} nK_{n}{}^{\left(0\right)}=\frac{K_{1}{}^{\left(0\right)}K_{n-1}{}^{\left(0\right)}}{{\langle e^{\beta \Delta U_{n}}\rangle }_{n}}\;. \end{array}$$

The brackets, ${\langle \ldots \rangle }_{n}$, indicate the thermal average utilizing configurations from the canonical simulation stream for the $W_{n}X$ cluster [15]. Here clustering is assessed in the same way on top and bottom: a clustered $W_{n}X$ configuration is analyzed as clustered configurations of $W_{n-1}X$ and WX. This amounts to an approximation if the $W_{n}X$ clusters are frequently ramified, i.e., branched. To see that point, suppose that the singlet W identified for Equation (5) is a node of the connectivity graph. Then the $W_{n-1}X$ remainder would not be connected. This concern is relieved for the more compact clusters of QCT applications, in contrast to the “Stillinger clusters” [19] for the physical cluster theory followed here.

In the energy combination of Equation (5), the rightmost term depends on the geometry of the W${}_{n}$X cluster sampled. The energies following that rightmost term are evaluated for a given conformation of W${}_{n}$X from the simulation stream for that cluster. One ligand (or each in turn) is distinguished to compose the energy difference suggested by the exchange

(7) $$WX+W_{n-1}X⇌X+W_{n}X\;.$$

Geometries of both species on the left of Equation (7) conform to the sampled W${}_{n}$X structure on the right. One understanding of $\Delta U_{n}$ (Equation (5)) is based on the following accounting. Consider first the combination $\left[U\left(WX\right)-U\left(X\right)\right]$ on the right side of Equation (5). This is the energy change for introducing one W ligand to a bare X ion. Next consider the remaining contribution $U\left(W_{n}X\right)-U\left(W_{n-1}X\right)$. This is the energy change for introducing an additional W ligand to a $W_{n-1}X$ complex. The difference $\Delta U_{n}$ thus reflects the crowding of the *n*th W ligand, including any effect of suboptimal binding of the *n*th W ligand to the X ion.

The free energies we arrive at correspond to water addition reactions (Equation (1)). They are experimentally measured by creating an ensemble with varying number of waters (*n*) in gas-phase mass spectrometry [45]. Because the cluster’s net charge remains constant as *n* varies, the energy combination of Equation (5) is not affected by the electrostatic potential of the phase [12].

For n=1, Equation (6) correctly reduces to the trivial case of $K_{0}{}^{\left(0\right)}=1$. In the evaluation of $K_{n}{}^{\left(0\right)}$ for n≥2, the value of $K_{1}{}^{\left(0\right)}$ can be supplied from experiment [45] or theory. This term incorporates the interaction strength between X and one W molecule. Carrying out subsequent steps in this scheme then addresses the issues that make anion hydration more challenging, i.e., competing H-bonding interactions of neighboring W molecules in those clusters.

The analysis above is based on the classical limit formula for the partition function $Q\left(W_{n}X\right)$. Thus, this approach does not directly address issues of quantum mechanical zero-point motion, except to the extent that a pragmatic external evaluation of $K_{1}^{\left(0\right)}$ incorporates zero-point motion empirically. This treatment of zero-point motion differs from the preceding harmonic approximation.

## 5. Conclusions

The rough-landscape analysis (Equation (6)) is surprisingly accurate compared to the available experiment data (Figure 2), even for the cases of ions that exhibit qualitative, though characteristic, anharmonic behaviors (Figure 1).

The tested rough-landscape remedy (Figure 5) to the harmonic approximation accounts for unfavorable effects due to crowding of the ligands. Though this rough-landscape procedure provides a distinctive correction, it is expected to break-down when split-shell structures predominate. From the technical perspective of QCT, n≳4 discrepancies are likely to be a consequence of a relaxed definition of clustering in the identification of ${\left(H_{2}O\right)}_{n}X$ clusters—including split-shell or ramified structures—that accompany *physical cluster theories.* With ramified structures, the central equation (Equation (6)) for the present rough-landscape approach can acquire some inconsistency. Further development of these physical cluster theories in the direction of QCT should remedy this issue, and should be the next step in this research direction.

## Data Availability

Data available from authors upon request.

## Abbreviations

QCT	Quasi-chemical theory
AIMD	*ab initio* molecular dynamics
